# NiYAl-Derived Nanoporous Catalysts for Dry Reforming of Methane

**DOI:** 10.3390/ma13092044

**Published:** 2020-04-27

**Authors:** Syota Imada, Xiaobo Peng, Zexing Cai, Abdillah Sani Bin Mohd Najib, Masahiro Miyauchi, Hideki Abe, Takeshi Fujita

**Affiliations:** 1 School of Environmental Science and Engineering, Kochi University of Technology, 185 Miyanokuchi, Tosayamada, Kami City, Kochi 782-8502, Japan; 245105w@gs.kochi-tech.ac.jp (S.I.); cai.zexing@kochi-tech.ac.jp (Z.C.); 2 National Institute for Materials Science, 1-1 Namiki, Tsukuba, Ibaraki 305-0044, Japan; peng.xiaobo@nims.go.jp (X.P.); abdillah.sani@nims.go.jp (A.S.B.M.N.); ABE.Hideki@nims.go.jp (H.A.); 3 School of Physics and Electronic Engineering, Xinyang Normal University, Xinyang 464000, China; 4 Tokyo Institute of Technology, 2-12-1 Ookayama, Meguro-ku, Tokyo 152-8552, Japan; mmiyauchi@ceram.titech.ac.jp

**Keywords:** nanoporous catalyst, methane dry reforming, NiYAl alloy, preferential oxidation, long-term durability

## Abstract

Dry reforming of methane can be used for suppressing the rapid growth of greenhouse gas emissions. However, its practical implementation generally requires high temperatures. In this study, we report an optimal catalyst for low-temperature dry reforming of methane with high carbon coking resistance synthesized from NiYAl alloy. A facile two-step process consisting of preferential oxidation and leaching was utilized to produce structurally robust nanoporous Ni metal and Y oxides from NiYAl_4_. The catalyst exhibited an optimal carbon balance (0.96) close to the ideal value of 1.0, indicating the optimized dry reforming pathway. This work proposes a facile route of the structural control of active metal/oxide sites for realizing highly active catalysts with long-term durability.

## 1. Introduction

Continuously increasing greenhouse gas (CO_2_) emissions trigger various climatic disasters and are gradually raising the sea level, which considerably decrease habitable areas. Therefore, developing a feasible method for the chemical or physical utilization of vast CO_2_ quantities represents an urgent task. Methane (CH_4_) is both a major component of natural gas and a greenhouse gas; hence, the dry reforming of methane (DRM, CH_4_ + CO_2_→2H_2_ + 2CO) could become a promising strategy for tackling excessive CO_2_ output without disrupting the current infrastructure and converting it to valuable chemical products [1]. However, this reaction requires a relatively high temperature (>800 °C) because of its endothermicity (ΔH°_298K_ = 247 kJ mol^−1^), which results in significant heat degradation due to material sintering. When DRM is performed in a low-temperature range from 400 to 600 °C, corresponding to low-temperature DRM (LT-DRM), its side reactions become more thermodynamically dominant leading to significant carbon coking due to methane decomposition (CH_4_→2H_2_ + C(s)) and the Boudouard reaction (2CO→CO_2_ + C(s)), that ultimately block the gas flow and cause a rupture of the reactor. Many heterogeneous (typically Ni-based) catalysts have been studied to date, and various modifications of the interactions between Ni atoms and oxide supports and/or structural design were considered [2,3,4,5,6,7,8,9]. To suppress a significant growth of carbon fabric on Ni particles, topological modification of the active sites located at the metal–oxide interface should be performed. Unlike the conventional nanoparticle–oxide supports, a well-connected metal–oxide topology obtained from bulk alloy via preferential oxidation [10,11,12] or leaching (dealloying) [13,14] was found to be a critical structural design with a highly active metal–oxide interface and long-term stability.

Here, we report nanoporous catalysts derived from NiYAl_x_ intermetallic precursors by combining preferential oxidation with leaching. As was shown in our previous study on binary NiY alloy [10], the preferential oxidation process resulted in the nanophase separation of Ni, Y oxide, and Al oxide in the precursor alloy, and the subsequent leaching process dissolved all Al components (Al and Al oxides) to yield a structurally robust nanoporous Ni/Y oxide catalyst. In the course of catalyst evaluation that involved intentional carbon accumulation, the obtained nanoporous catalyst was more resistant to carbon deposition than the conventional Ni-based (Ni/Al_2_O_3_) and Raney Ni catalysts for LT-DRM. In contrast to conventional chemical routes, the top-down process starting from bulk alloy can produce advanced materials with high catalytic activity and coking tolerance for methane conversion.

## 2. Materials and Methods

### 2.1. Preparation of Nanoporous Catalysts

**Figure 1:**
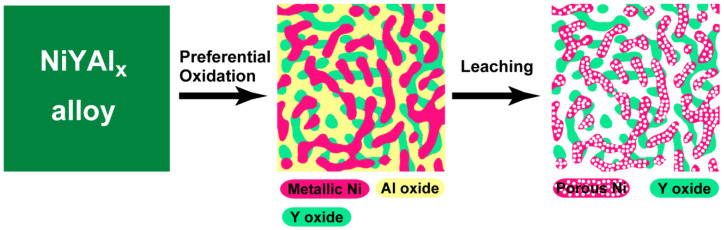
Schematic illustration of the catalyst preparation from NiYAl_x_ alloys.

The utilized catalyst fabrication route is outlined in Figure 1. 

Ni_33.3_Y_33.3_Al_33.3_, Ni_25_Y_25_Al_50_, and Ni_16.7_Y_16.7_Al_66.6_ (at.%) were selected as intermetallic precursors for NiYAl, NiYAl_2_ and NiYAl_4_, respectively, using the Ni–Al–Y ternary phase diagram [15]. Ingots were prepared by melting pure Ni, Y, and Al metals (>99.9 at.%) inside an Ar-protected arc melting furnace. The resulting Ni–Y–Al alloy ingots were ground in a mortar and sieved to obtain powder precursors with average particle sizes of 50–60 μm. During the preferential oxidation process, the Ni–Y–Al alloy precursors were heated in a gas stream consisting of CO (2 vol.%), O_2_ (1 vol.%), and Ar (97 vol.%) at a flow rate of 60 mL min^−1^ and temperature of 873 K for 12 h to obtain phase-separated Ni–Y_2_O_3_–Al_2_O_3_ composites. To perform acid leaching, these composites (~0.5 g) were autoclaved in a 15 M NaOH solution at a pressure of 5 atm and temperature of 150 °C for 6 h to dissolve Al, followed by thorough rinsing with water and drying under air.

### 2.2. Preparation of Conventional Ni Catalysts

Ni/Al_2_O_3_ composite was prepared by a conventional impregnation method. Following the dissolution of Ni(NO_3_)_2_∙6H_2_O (0.8 g, Sigma-Aldrich, Louis, MO, USA) in ethanol (20 mL), Al_2_O_3_ powder (0.3 g, Sigma-Aldrich Louis, MO, USA) was added to the reaction solution. The resulting mixture was stirred for 8 h, after which ethanol was removed by evaporation at 353 K. Ni/Al_2_O_3_ catalyst was synthesized through the calcination of the obtained product in an H_2_–Ar gas mixture (5 vol.% H_2_) at 873 K for over 4 h.

To obtain Raney Ni catalyst, commercial Ni–Al (50/50 wt.%) precursor powder was purchased from Kojundo Chemical Laboratory CO., Ltd., Saitama, Japan. Approximately 0.5 g of this powder was dealloyed in a 30 wt.% NaOH (97% Wako, Japan) solution for 4 h at 50 °C, rinsed thoroughly with water, and dried under air.

### 2.3. Microstructural Characterization

Microstructures of the obtained catalysts were characterized by scanning transmission electron microscopy (STEM, JEM-2100F, JEOL, Tokyo, Japan) and energy-dispersive x-ray spectroscopy (EDS, Ince Energy TEM 250, Oxford, Abingdon, UK). The analyzed samples were transferred onto a Cu grid without using a uniform carbon support film. X-ray diffraction (XRD) profiles were recorded using a Rigaku SmartLab X-ray diffractometer (Rigaku, Tokyo, Japan) with Cu Kα radiation (40 kV). Surface morphologies were observed by scanning electron microscope (SEM, Hitachi SU-8030, Tokyo, Japan) at an accelerating voltage of 15 kV. The deposited carbon present after the DRM process was evaluated using a thermal gravimetric-differential thermal analyzer (TG-DTA, NETZSCH, STA 2500, Selb, Germany) under air. The sharp mass loss above 500 °C corresponded to the combustion of carbon.

### 2.4. Catalytic Studies

LT-DRM was conducted inside a fixed-bed flow reactor with an inner diameter of 10 mm. A sample with a mass of 0.1 g was loaded into the reactor at 550 °C in a gas mixture of CH_4_ (10 mL/min), CO_2_ (10 mL/min), and N_2_ (5 mL/min) with a total flow rate of 25 mL min^−1^ to accelerate the carbon accumulation process. The composition of the effluent gas was monitored by a gas chromatograph (Shimadzu, TCD, GC-8A) with a column made of activated charcoal. The formulas utilized for calculating consumption rates, formation rates, conversions, and the H_2_/CO ratio are provided below [13]:

$${\mathrm{CH}}_{4}\;\mathrm{conv}.\;[\%]=\frac{{\left[{\mathrm{CH}}_{4}\right]}_{\mathrm{in}}-{\left[{\mathrm{CH}}_{4}\right]}_{\mathrm{out}}}{{\left[{\mathrm{CH}}_{4}\right]}_{\mathrm{in}}}\times 100$$

$${\mathrm{CO}}_{2}\;\mathrm{conv}.\;[\%]=\frac{{\left[{\mathrm{CO}}_{2}\right]}_{\mathrm{in}}-{\left[{\mathrm{CO}}_{2}\right]}_{\mathrm{out}}}{{\left[{\mathrm{CO}}_{2}\right]}_{\mathrm{in}}}\times 100$$

CH_4_ consumption rate = [CH_4_]_in_ flow rate × CH_4_ conv.

CO_2_ consumption rate = [CO_2_]_in_ flow rate × CO_2_ conv.

$$H_{2}\;\mathrm{formation}\;\mathrm{rate}=[{\mathrm{CH}}_{4}{]}_{\mathrm{in}}\;\mathrm{flow}\;\mathrm{rate}\;\times \;\frac{{\left[H_{2}\right]}_{\mathrm{out}}}{{\left[{\mathrm{CH}}_{4}\right]}_{\mathrm{in}}}$$

$$\mathrm{CO}\;\mathrm{formation}\;\mathrm{rate}=({\left[{\mathrm{CH}}_{4}\right]}_{\mathrm{in}}\;\mathrm{flow}\;\mathrm{rate}+{\left[{\mathrm{CO}}_{2}\right]}_{\mathrm{in}}\;\mathrm{flow}\;\mathrm{rate})\;\times \;\frac{{\left[\mathrm{CO}\right]}_{\mathrm{out}}}{{\left[{\mathrm{CH}}_{4}\right]}_{\mathrm{in}}+{\left[{\mathrm{CO}}_{2}\right]}_{\mathrm{in}}}$$

$$H_{2}/\mathrm{CO}\;\mathrm{ratio}=\frac{H_{2}\;\mathrm{formation}\;\mathrm{ratio}}{\mathrm{CO}\;\mathrm{formation}\;\mathrm{ratio}}$$

where […]_in_ and […]_out_ represent the molar concentrations in the feed gas and effluent gas, respectively.

## 3. Results and Discussion

**Figure 2:**
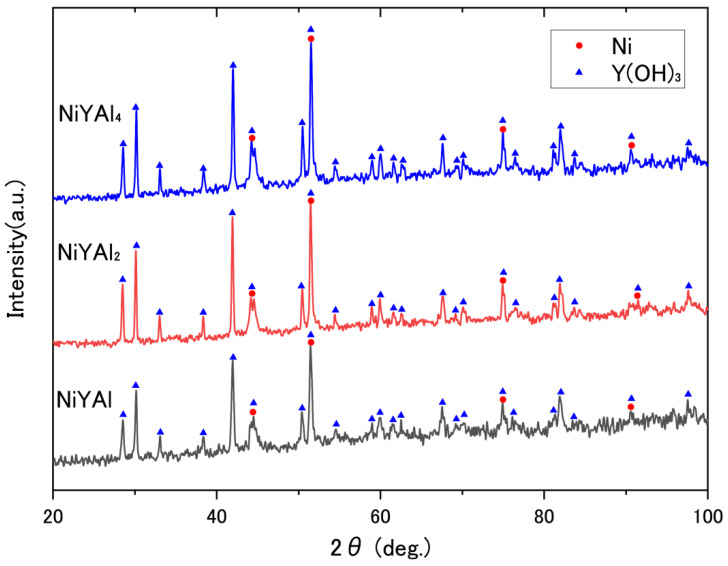
X-ray diffractograms of the nanoporous catalysts obtained from NiYAl_4_, NiYAl_2_, and NiYAl intermetallic precursors.

Intermetallic precursors were obtained by arc-melting, and the resulting NiYAl_4_ (JCPDF#50-1236), NiYAl_2_ (#76-8082), and NiYAl (#22-0008) compounds were identified in the as-made precursor alloys by XRD, as shown in Figure S1. The subsequent preferential oxidation via the CO + O_2_ reaction induced the phase separation of Ni, Al_2_O_3_ (#04-0877), Y_2_O_3_ (#43-0661), and residual intermetallics according to Figure S2. After conducting high-pressure leaching to remove Al components, the resultant products were ultimately converted to Ni and Y(OH)_3_ (Figure 2); the latter was subsequently converted to Y_2_O_3_ through a catalytic reaction in the next step. The conversion from Y(OH)_3_ to Y_2_O_3_ was driven by the DRM reaction as well as heat treatment [13,16].

**Figure 3:**
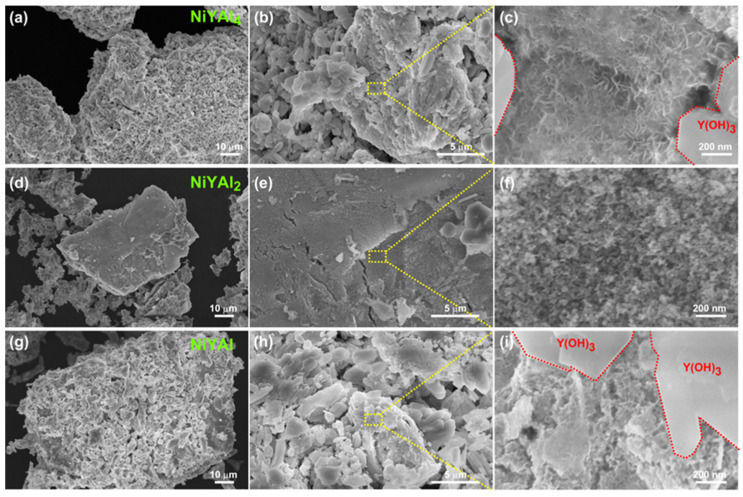
SEM images of the nanoporous catalysts derived from (**a**–**c**) NiYAl_4_, (**d**–**f**) NiYAl_2_, and (**g**–**i**) NiYAl intermetallic precursors.

The obtained scanning electron microscope (SEM) images are displayed in Figure 3. 

**Figure 4:**
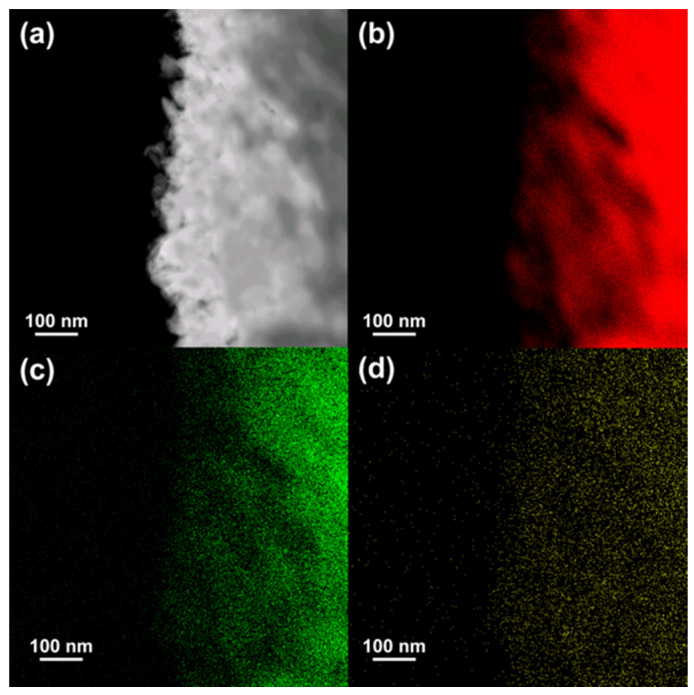
(**a**) STEM image and (**b**–**d**) energy-dispersive x-ray spectroscopy (EDS) chemical maps of the nanoporous Ni region in the NiYAl_4_-derived sample showing the distributions of Ni (red), Y (green), and O (yellow) elements.

The microstructures of the NiYAl- and NiYAl_4_-derived samples contain mixed but distinct regions of squarish Y(OH)_3_ and nanoporous Ni. The microstructure of the NiYAl_2_-derived catalyst includes the nanoporous Ni and isolated Y(OH)_3_ regions. The nanoporous Ni region in the NiYAl_4_-based sample was analyzed by STEM–EDS, as shown in Figure 4. 

Its average pore size was approximately 30 nm, while the composition of the nanoporous Ni region in the sample, represented by the formula 91.9Ni–2.6Y–0.5Al–5O (at.%), was relatively uniform.

**Figure 5:**
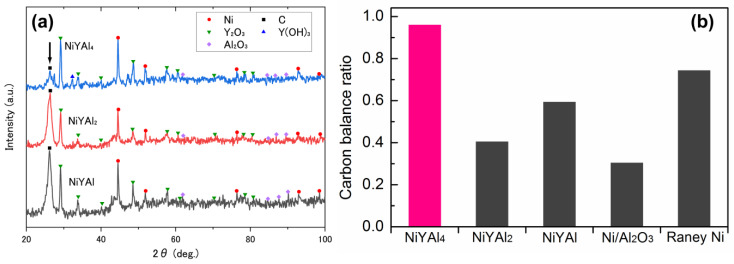
(**a**) X-ray diffractograms of the spent catalysts prepared from NiYAl_4_, NiYAl_2_, and NiYAl intermetallic precursors. The arrow indicates the position of the carbon peak. (**b**) Carbon balance ratio for the present catalysts and reference catalysts. Carbon balance = {CO formation rate/(CH_4_ consumption rate + CO_2_ consumption rate)}.

The catalytic properties of the nanoporous catalysts produced from NiYAl, NiYAl_2_, and NiYAl_4_ precursors are summarized in Table 1. Here, the conventional Ni/Al_2_O_3_ and Raney Ni catalysts serve as references. The H_2_/CO ratio is an important indicator of the coking resistance (its ideal value is equal to one, while higher magnitudes of this parameter indicate a significant coking effect). Photographs of the initial and spent catalyst samples are shown in Figure S3. As expected from the increased volume in Figure S3, the spent NiYAl_2_-derived sample demonstrated the most intense coking effect (H_2_/CO ratio = 2.4), and the NiYAl_4_-derived catalyst exhibited the weakest coking effect (H_2_/CO ratio = 0.7). The XRD analysis (Figure 5a) reveals that the spent samples are composed of Ni and Y_2_O_3_ converted from Y(OH)_3_. In addition, the carbon peaks with the highest intensity appear for the NiYAl_2_-derived catalyst, whereas the smallest carbon peaks are observed for the NiYAl_4_-derived one. The NiYAl_4_-base sample exhibited no catalytic activity after the preferential oxidation (without etching), indicating that the final leaching process was an important step toward achieving the optimal catalyst microstructure. The conventional Ni/Al_2_O_3_ and Raney Ni catalysts also demonstrated strong coking effects (their H_2_/CO ratios were 2.3 and 1.4, respectively). We calculated the carbon balance for these catalysts; carbon balance = {CO formation rate/(CH_4_ consumption rate + CO_2_ consumption rate)}, and the results are shown in Figure 5b. The conventional catalyst Ni/Al_2_O_3_ exhibits the lowest carbon balance of 0.31. Although Raney Ni shows a relatively higher carbon balance (0.74), the NiYAl_4_-derived sample represented the optimal carbon balance (0.96) close to the ideal value of 1.0, indicating the optimal DRM pathway. The thermal gravimetric analysis (Figure S4) confirms that the spent NiYAl_4_-derived sample exhibit the lowest amount of coking.

## 4. Conclusions

In this study, we fabricated nanoporous catalysts from NiYAl_x_ intermetallic precursors by a combined process of preferential oxidation and leaching. The optimal catalyst obtained from NiYAl_4_ possessed a structurally robust nanoporous Ni metal and Y oxide structure with high coking resistance for LT-DRM. The described facile fabrication process can be utilized for fabricating highly active catalysts with long-term stability from various binary, ternary, and quaternary metal alloys.

## Figures and Tables

**Table 1:** Dry reforming of methane (DRM) parameters of various Ni catalysts including the nanoporous catalysts obtained from NiYAl, NiYAl_2_, and NiYAl_4_ intermetallic precursors in this study as well as conventional Ni/Al_2_O_3_ and Raney Ni catalysts. The NiYAl_4_ sample showed no catalytic activity after the preferential oxidation (CO + O_2_) without leaching.

Sample	CH_4_ Conv. (%)	CO_2_ Conv. (%)	CH_4_ Consumption Rate (mmol h^−1^)	CO_2_ Consumption Rate (mmol h^−1^)	H_2_ Formation Rate (mmol h^−1^)	CO Formation Rate (mmol h^−1^)	H_2_/CO Ratio
NiYAl_4_	12	19	3.3	5.1	5.7	8.1	0.7
NiYAl_2_	45	33	12	8.8	18	8.5	2.2
NiYAl	32	29	8.5	7.9	14	9.8	1.4
NiYAl_4_ (CO + O_2_)	0	0	-	-	-	-	-
Ni/Al_2_O_3_	56	37	15	10	18	7.7	2.3
Raney Ni	33	31	8.9	8.5	17	13	1.4

Reaction conditions: 0.1 g; 550 °C; CH_4_ (10 mL/min), CO_2_ (10mL/min), and N_2_ (5 mL/min) with a total flow rate of 25 mL min^−1^; 4–6 h.

## Supplementary Materials

The following are available online at https://www.mdpi.com/1996-1944/13/9/2044/s1, Figure S1: X-ray diffractograms of NiYAl4, NiYAl2, and NiYAl intermetallic precursors, Figure S2: X-ray diffractograms of the NiYAl4, NiYAl2, and NiYAl samples obtained after the preferential oxidation with CO + O2 gas mixture, Figure S3: (left) Photograph of the initial and spent NiYAl-derived catalyst. The degree of carbon coking was estimated from the increase in volume between the initial and spent samples. (right) Photograph of the spent NiYAl-, NiYAl2-, and NiYAl4-derived catalysts. The initial NiYAl2- and NiYAl4-derived samples were similar in appearance to the initial NiYAl-derived catalyst. Figure S4: TG analysis of the spent catalysts derived from NiYAl, NiYAl_2_, and NiYAl_4_.
