# Supplementary material for: NiYAl-Derived Nanoporous Catalysts for Dry Reforming of Methane
Source: Materials (Basel). 2020 Apr 27;13(9):2044. doi: 10.3390/ma13092044 (PMC7254345; doi:10.3390/ma13092044)
Supplement: Supplementary file 1 [file materials-13-02044-s001.pdf]

materials

Supplementary Materials

NiYAl-Derived Nanoporous Catalysts for Dry Reforming of Methane

Syota Imada [1], Xiaobo Peng [2], Zexing Cai [1,3], Abdillah Sani Bin Mohd Najib [2], Masahiro Miyauchi [4], Hideki Abe [2] and Takeshi Fujita [1,*]

1 School of Environmental Science and Engineering, Kochi University of Technology, 185 Miyanokuchi, Tosayamada, Kami City, Kochi 782-8502, Japan; 245105w@gs.kochi-tech.ac.jp (S.I.); cai.zexing@kochi-tech.ac.jp (Z.C.)
2 National Institute for Materials Science, 1-1 Namiki, Tsukuba, Ibaraki 305-0044, Japan; peng.xiaobo@nims.go.jp (X.P.); abdillah.sani@nims.go.jp (A.S.B.M.N.); ABE.Hideki@nims.go.jp (H.A.)
3 School of Physics and Electronic Engineering, Xinyang Normal University, Xinyang 464000, China
4 Tokyo Institute of Technology, 2-12-1 Ookayama, Meguro-ku, Tokyo 152-8552, Japan; mmiyauchi@ceram.titech.ac.jp
* Correspondence: fujita.takeshi@kochi-tech.ac.jp

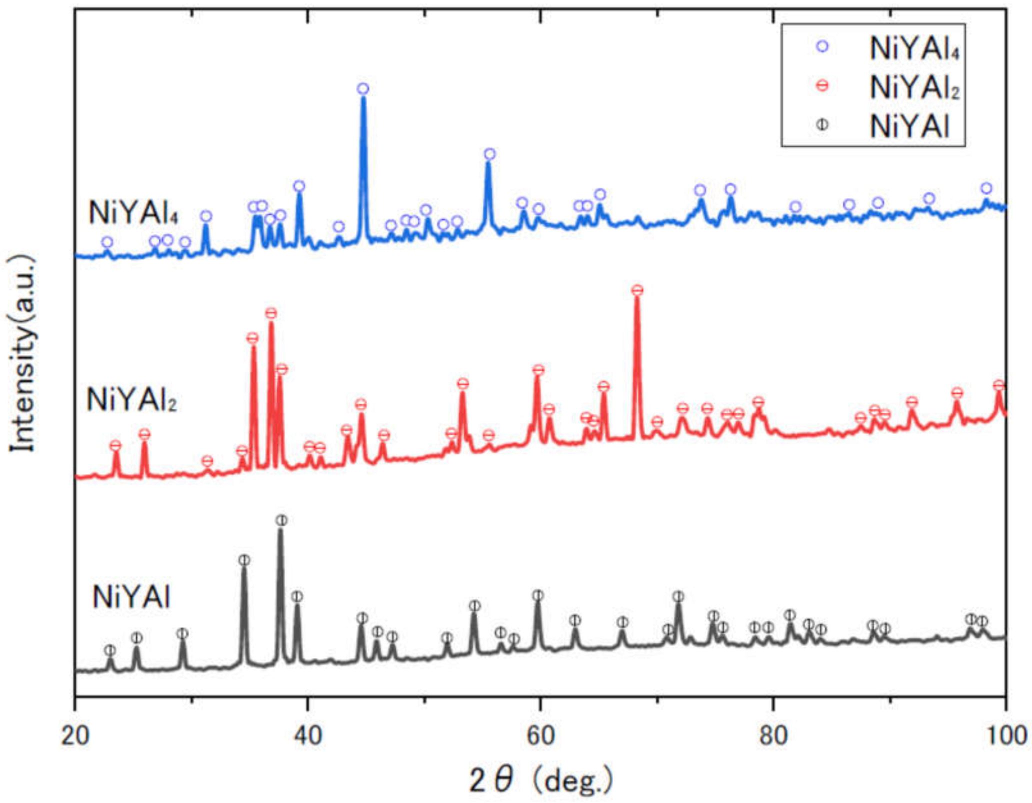

Figure S1. X-ray diffractograms of $NiYAl_4$, $NiYAl_2$, and NiYAl intermetallic precursors.

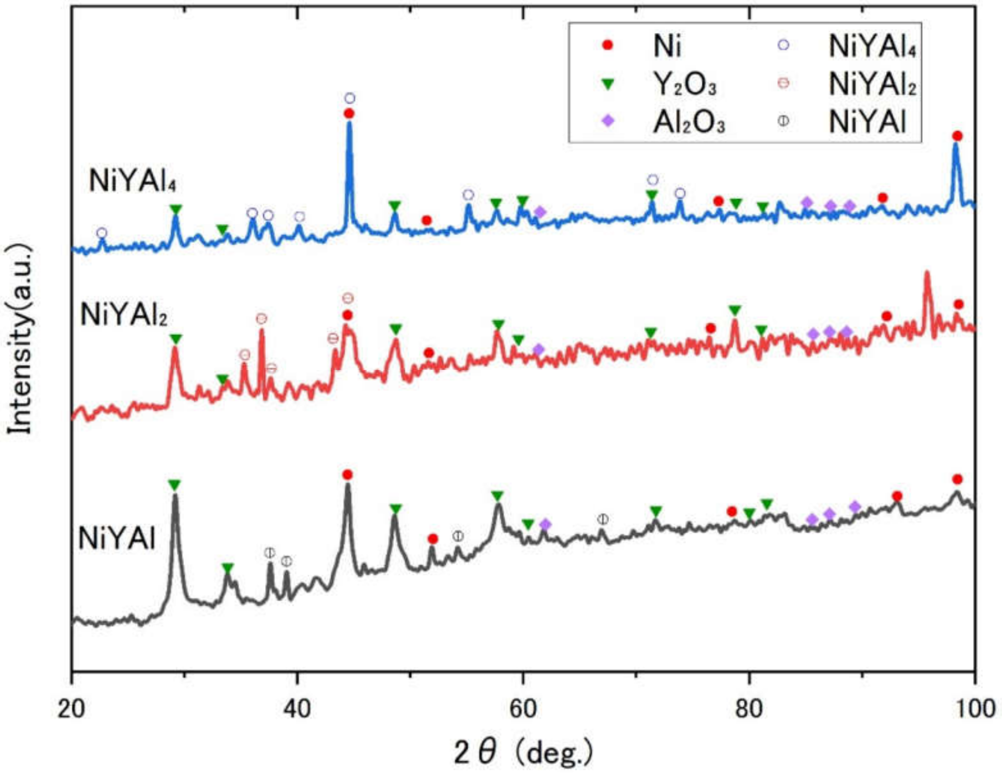

Figure S2. X-ray diffractograms of the $NiYAl_4$, $NiYAl_2$, and NiYAl samples obtained after the preferential oxidation with $CO + O_2$ gas mixture.

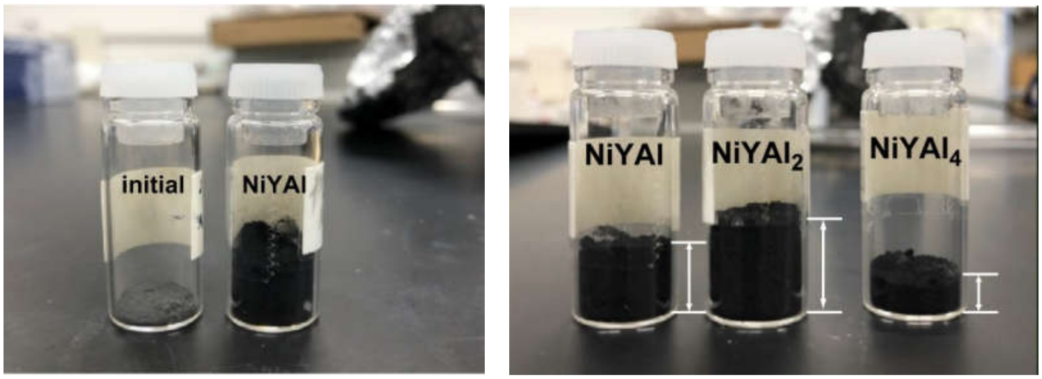

Figure S3. (left) Photograph of the initial and spent NiYAl-derived catalyst. The degree of carbon coking was estimated from the increase in volume between the initial and spent samples. (right) Photograph of the spent NiYAl-, $NiYAl_2$-, and $NiYAl_4$-derived catalysts. The initial $NiYAl_2$- and $NiYAl_4$-derived samples were similar in appearance to the initial NiYAl-derived catalyst.

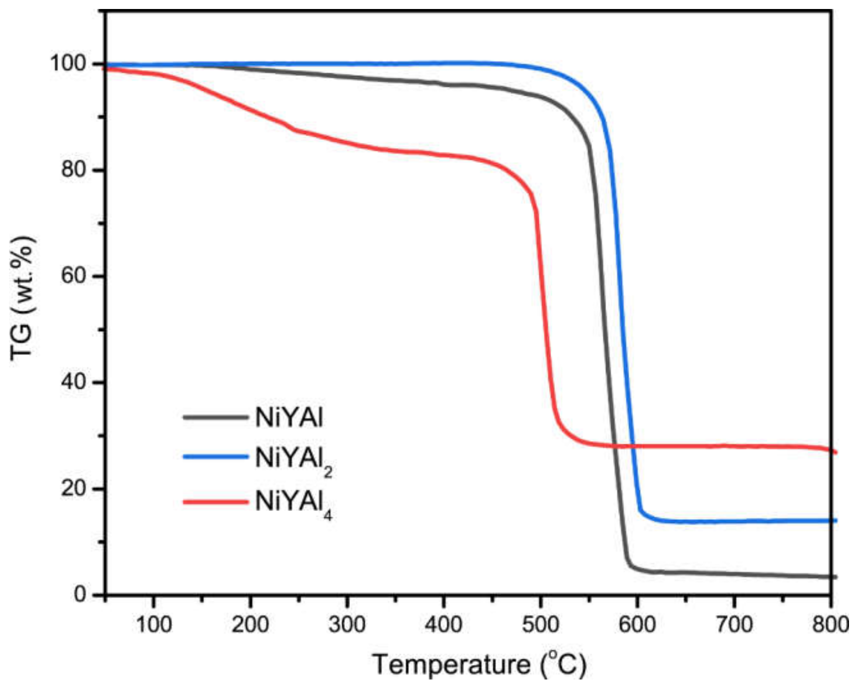

Figure S4. TG analysis of the spent catalysts derived from NiYAl, $NiYAl_2$, and $NiYAl_4$.

© 2020 by the authors. Licensee MDPI, Basel, Switzerland. This article is an open access article distributed under the terms and conditions of the Creative Commons Attribution (CC BY) license (http://creativecommons.org/licenses/by/4.0/).
